# Evaluation of the effect of Muller muscle-conjunctival resection on meibomian glands by meibography

**DOI:** 10.5935/0004-2749.2024-0029

**Published:** 2024-09-16

**Authors:** Fatma Savur, Muzaffer Said Güler

**Affiliations:** 1 Department of Ophthalmology, Basaksehir Cam and Sakura City Hospital, University of Health Sciences, Istanbul, Turkey

**Keywords:** Meibomian glands, Blepharoptosis, Preoperative period, Conjunctiva, Muscles, Eyelid diseases, Diagnostic techniques, ophthalmological

## Abstract

**Purpose:**

To evaluate the effect of upper eyelid ptosis repairwith Muller muscle-conjunctival resection on meibomian gland function and ocular surface parameters.

**Methods:**

Thirty-eight patients who underwent ptosis repair with Muller muscle-conjunctival resection were retrospectively reviewed. Meibomian gland loss, Ocular Surface Disease Index OXFORD score, meiboscore, and noninvasive keratograph break-up time were measured preoperatively and at 1st, 3rd, and 6th months postoperatively.

**Results:**

Noninvasive keratograph break-up time values decreased significantly at 1st and 3rd months postoperatively compared to the preoperative level, but were similar to the preoperative level at 6th months postoperatively (p<0.001 and p=0.628, respectively). Ocular surface disease index, OXFORD score, meibomian gland loss, and meiboscore values increased significantly in the 1st and 3rd postoperative months compared to the preoperative period, but these values decreased to preoperative levels in the 6th postoperative month (p<0.001 and p>0.05, respectively).

**Conclusion:**

There is a transient deterioration in meibography findings and OSDI score in the early postoperative period afterMuller muscle-conjunctival resection. Patients undergoing Muller muscle-conjunctival resection may require topical lubricants, especially in the first 3 postoperative months.

## INTRODUCTION

Currently, the most commonly used techniques for the treatment of ptosis are levator supports (anterior approach) and Muller muscle-conjunctival resection (MMCR) (posterior approach). The Fasanella-Servat procedure was a simple vertical eyelid shortening procedure to correct mild ptosis with a tarsus-müller muscle resection, originally described as levator and tarsal resection with a posterior approach^(^1^)^. Putterman and Urist developed a modification of the Fasanella-Servat operation known as MMCR, in which the tarsus is preserved by excising the Muller muscle alone without damaging the tarsal plate^(^2^)^. Before performing MMCR, it is imperative to perform a 2.5% or 10% phenylephrine test and confirm elevation of the ptotic eyelid. MMCR has been a reliable procedure for ptosis correction since its first description by Putterman and Urist. Over the years, various modifications to the procedure and algorithms for tissue removal have been reported^(^2^,^3^,^4^,^5^)^. However, the extent of tissue removal varies depending on the operating surgeon’s technique. Another prerequisite for surgery is the adequacy of the healthy conjunctiva in the superior fornix. However, there is no clear consensus among surgeons regarding the effect of MMCR on the ocular surface. The palpebral conjunctiva contains goblet cells and accessory lacrimal glands of Krause and Wolfring which play an important role in tear film composition. Whether the excision of conjunctival tissue, including these structures, during the MMCR procedure would lead to tear film instability has been a major concern and an important research topic^(^6^)^. In previous studies, the dry eye assessment questionnaire, Schirmer test, tear break-up time, and fluorescein staining tests were used to evaluate tear film stability^(^6^)^.

In the present study, we aimed to evaluate the ocular surface changes occurring in the postoperative period after MMCR and to detect possible meibomian gland loss using meibography. To the best of our knowledge, this is the first study to evaluate meibomian gland changes by meibomiography in patients undergoing MMCR.

## METHODS

Thirty-eight eyes of 38 patients who underwent conjunctival mullerectomy for ptosis by a single surgeon (FS) at a single center between January 2021 and May 2023 were retrospectively analyzed. The study was approved by the Institutional Review Board and complied with the principles enshrined in the Declaration of Helsinki. All patients had good levator function (≥8 mm). Patients with a history of previous ocular, orbital, eyelid, or eyebrow surgery, trauma, conjunctival and ocular surface problems, and any systemic disease that may affect eyelid position were excluded from the study. No additional surgical procedure was performed in all patients. Preoperative and postoperative marginal reflex distance 1 (MRD1) measurements, Schirmer test, tear film breakage time, fluorescein staining, and meibiography measurements were evaluated. Meibomiography measurements and ocular surface disease index (OSDI) scores were performed and evaluated by the same surgeon (MSG).

None of the patients had postoperative keratopathy, eyelid contour disorder, or other complications. Preoperative measurements and measurements performed at postoperative 1st, 3rd, and 6th months were used for the analysis.

The extent of resection of conjunctival mullerectomy was determined according to phenylephrine test results. The response was evaluated 5 minutes after the instillation of 2.5% phenylephrine. If the desired ptosis correction resulted in an inadequate response in the phenylephrine test, a 10 mm resection was performed. Nine-millimeter excision was performed in moderate responders, 8-mm resection in case of extreme response, and 11-mm resection in unresponsive patients.

### Measurements and evaluations

**Figure 1:**
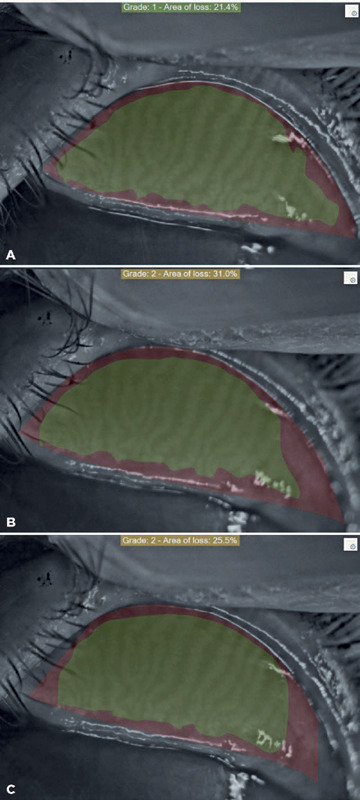
The percentage loss of meibomian glands was calculated automatically using the Phoenix software module. A) Preoperative meibography image; B: Grade 2 loss at 1st month postoperatively; C: Grade 2 loss at 6th month postoperatively.

Meibography of the upper lid of each eye was performed. Analysis and markings were made by the same ophthalmologist (M.S.G) using the Sirius corneal topography device and its proprietary Phoenix imaging software module (C.S.O, Costruzione Strumenti Oftalmici, Florence, Italy). Loss amounts were calculated in percentage (%) and according to the rating system. The Phoenix software provided measurements of dropout percentage, along with categorized dropouts utilizing a scale within the area. This scale was highlighted using the users’ manual tool, according to the grading system. The grading system used was as follows: no loss = grade 0; <25% loss = grade 1; 26%-50% loss = grade 2; 51%-75% loss = grade 3; and >75% loss = grade 4. At least five separate meibography images were obtained for each patient. Among these five meibography images, the amount of Meibomian glands (MG) loss was recorded separately on the three images with the best contrast and image quality (Figure 1). Then, the average value of the three meibography images for each eyelid was used in statistical analysis.

The noninvasive keratograph break-up time (N1KBUT) test was also performed with the Sirius topography device. Videokeratoscopy in the topography device produces quantitative results such as NIKBUT by analyzing information obtained at up to 25 frames per second from more than 400 film frames created using images reflected from the corneal surface.

Subjective ocular symptoms were assessed using the Ocular Surface Disease Index. The OSD1 is a 12-item questionnaire designed for a rapid assessment of symptoms of ocular discomfort consistent with dry eye disease. OSDI enables a convenient, rapid, and reliable diagnosis of ocular surface disease and evaluates ocular findings associated with dry eye disease. OSDI scores between 0 and 12 are considered normal, while OSDI scores of ≥13 are considered abnormal.

### Surgical method

**Figure 2:**
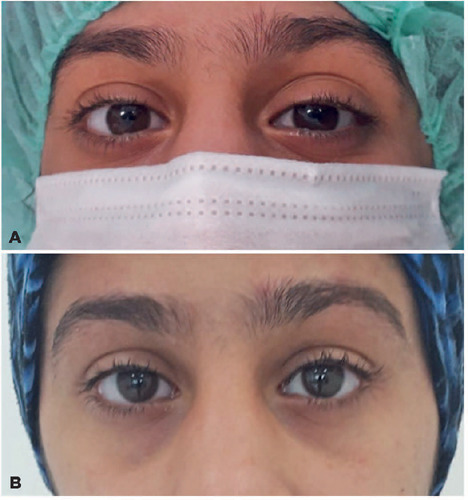
Preoperative (A) and 6th-month postoperative photograph (B) of a patient after Muller muscle-conjunctival resection.

After the necessary surgical site preparation, topical anesthesia was applied with proparacaine hydrochloride 0.5% eye drops. The eyelid margin was marked with a marking pen parallel to the medial and lateral limbus levels. The upper eyelid was reversed with the Desmarres retractor. Marking was made on the upper eyelid tarsal border parallel to the markings on the eyelid margin. Based on these marks, marking was made in the conjunctival area from a distance of half the planned MMCR amount. Subconjunctival 2% lidocaine containing 1:100,000 epinephrine was applied to the marking area. The same anesthetic was injected subcutaneously medially and laterally to the lid fold line, at the planned site of protrusion of the sutures above the skin. Traction sutures were passed from the conjunctival marking area with 4-0 silk, covering only the conjunctiva and Muller muscle. The conjunctiva and Muller muscle were separated from the underlying levator aponeurosis by applying traction to the sutures. Then, the Putterman Mullerectomy clamp was placed to prevent the entry of the tarsus into the clamp. Then, it was sutured from one end to the other end horizontally and straight with a 6-0 polypropylene suture under the clamp. The suture was passed through the entire thickness of the eyelid and removed from the skin. The conjunctival surface was reentered by placing a booster in between to prevent abrasion of the skin surface. The suture was then recrossed from one end of the wound to the other in the opposite direction, horizontally, and reextracted to the skin surface. The clamped conjunctiva and Muller muscle were resected with a no. 15 scalpel, taking care not to include the levator aponeurosis and not to interrupt the continuous suture. The operation was terminated by placing a booster between the suture ends and knotting them on the skin. The eyelid was inverted and the eye was closed with antibiotic ointment for one day. Patients were advised to apply ice compresses to their eyelids for the first 48 hours after surgery and to use eye drops containing a combination of topical loteprednol etabonate and tobramycin before going to bed for one week. Conjunctival sutures were removed one week after surgery. Preoperative and postoperative 3rd and 6th-month photographic records were obtained for all patients. Representative photographs of a patient are presented in figure 2.

### Statistical methods

Continuous variables were presented as mean ± standard deviation or median (minimum, maximum), while categorical variables were presented as frequency (percentage). The normality of the distribution of continuous variables was assessed using the Kolmogorov-Smirnov test. Wilcoxon test was used for the repeated measurement analysis. SPSS software (IBM SPSS Statistics for Windows, Version 28.0; Armonk, NY, IBM Corp.) was used for statistical analyses. P-values <0.05 were considered indicative of statistical significance.

## RESULTS

The mean age of the patients was 34.4 ± 12.8 (range, 19-60). Out of the 38 patients, 34 (89.5%) were female and 4 (10.5%) were male. Nineteen (50%) patients had ptosis in their right eye and 19 (50%) in their left eye (Table 1). The mean NIKBUT value showed a significant decrease in the postoperative 1st and 3rd months compared to the preoperative value (p<0.001). Mean OSDI, OXFORD score, MGL, and Meiboscor values increased significantly at 1 and 3 months postoperatively compared to the preoperative levels (p<0.001). The NIKBUT value at the postoperative 6th month was significantly higher than that at the postoperative 1st and 3rd months. The OSDI, OXFORD score, MGL, and Meiboscor at postoperative 6th month were significantly lower than that postoperative 1st and 3rd months (p<0.001 for all). NIKBUT, OSDI, OXFORD score, MGL and Meiboscor values measured at the 6th postoperative month were comparable to the corresponding preoperative levels (p=0.628, p = 0.171,p = 0.073,p=0.056, and p = 0.056, respectively) (Table 2).

**Table 1:** Demographic characteristics and parameters of the study population

		Median value (Min-Max)	Mean ± SD/n (%)
Age (years)		35.0 (19.0-60.0)	34.4 ± 12.8
Laterality, n (%)	Left		19 (50.0)
Right			19 (50.0)
Sex, n (%)	Female		34 (89.5)
	Male		4 (10.5)
**Preoperative**			
NIKBUT		17.0 (10.0-17.0)	15.3 ± 2.4
OSDI		20.0 (8.0-30.6)	18.9 ± 6.7
OXFORD		0.00 (0.00-1.00)	0.42 ± 0.5
Meiboscore (GradeI		1.00(0.00-2.00)	0.84 ± 0.4
MGL (%)		13.6 (8.6-28.9)	15.8 ± 6.2

OSDI= ocular surface disease index; MGL= meibomian gland loss.

**Table 2:** Comparison of ocular surface parameters and meibography measurement values between preoperative and postoperative 1st, 3rd, and 6th months

	Median value (Min-Max)	Mean±SD	p*	P**	p***
**NIKBUT**					
Preoperative	17.0 (10.0-17.0)	15.3 ± 2.4			
Postoperative 1 month	10.0 (4.0-17.0)	9.8 ± 3.3	**0.000** ^w^		
Postoperative 3 month	13.0 (8.0-18.0)	13.2 ± 2.7	**0.000** ^w^	**0.000** ^w^	
Postoperative 6 month	15.0 (10.0-17.0)	14.6 ± 2.4	0.628 ^w^	**0.000** ^w^	**0.000** ^w^
**OSDI**					
Preoperative	20.0 (8.0-30.6)	18.9 ± 6.7			
Postoperative 1 month	39.5 (16.0-46.0)	36.5 ± 7.5	**0.000** ^w^		
Postoperative 3 month	26.0 (12.0-36.7)	25.9 ± 6.3	**0.000** ^w^	**0.000** ^w^	
Postoperative 6 month	20.0 (10.0-31.0)	19.1 ± 6.1	0.171 ^w^	**0.000** ^w^	**0.000** ^w^
**OXFORD**					
Preoperative	0.00 (0.00-1.00)	0.42 ± 0.50			
Postoperative 1 month	2.00 (0.00-2.00)	1.63 ± 0.59	**0.000** ^w^		
Postoperative 3 month	1.00 (0.00-2.00)	1.05 ± 0.61	**0.000** ^w^	**0.000** ^w^	
Postoperative 6 month	1.00 (0.00-2.00)	0.66 ± 0.53	0.063 ^w^	**0.000** ^w^	**0.000** ^w^
**Meiboscore (Grade)**					
Preoperative	1.00 (0.00-2.00)	0.84 ± 0.49			
Postoperative 1 month	2.00 (1.00-3.00)	1.89 ± 0.45	**0.000** ^w^		
Postoperative 3 month	1.00 (0.00-2.00)	1.29 ± 0.61	**0.000** ^w^	**0.000** ^w^
Postoperative 6 month	1.00 (0.00-2.00)	0.58 ± 0.59	0.056 ^w^	**0.000** ^w^	**0.000** ^w^
**MGL (%)**					
Preoperative	13.6 (8.6-28.9)	15.8 ± 6.2			
Postoperative 1 month	29.6 (21.6-56.7)	33.5 ± 9.2	**0.000** ^w^		
Postoperative 3 month	24.2 (10.0-92.0)	24.8 ± 12.8	**0.000** ^w^	**0.000** ^w^	
Postoperative 6 month	15.0 (9.0-29.0)	16.2 ± 5.6	0.073 ^w^	**0.000** ^w^	**0.000** ^w^

OSDI= ocular surface disease index; ^w^Wilxocon test.

*versus preoperative; **versus postoperative 1 month; ***versus postoperative 3 month.

## DISCUSSION

The eyelids are directly responsible for protecting and lubricating the eye. Therefore, after any eyelid surgery, the ocular surface is liable to be affected by both anatomical and functional changes in the eyelid and postoperative inflammation. The tear film is described as a three-layered structure, with an internal mucous layer in contact with the cornea epithelium, an aqueous intermediate layer forming the bulk of the tear volume, and a lipid outer layer that prevents evaporation of the tears. The mucous layer is produced by both the goblet cells and the corneal and conjunctival epithelia. The aqueous layer is produced both by the main lacrimal gland and by the accessory lacrimal glands of Krause and Wolfring. The outer lipid layer is secreted mainly by the meibomian glands and in part also by the glands of Moll and Zeiss. MMCR is a safe and effective surgery to correct ptosis in patients with intact levator muscle function and a positive preoperative response to the phenylephrine test. Although some modifications have been suggested over the years, the basic surgical procedure involves resection of the Muller muscle and conjunctiva, followed by suturing the conjunctiva and Müller muscle to the Tarsus, as described by Putterman and Urist^(^2^)^. The authors found that during preoperative surgical planning, further corrections can be made when a strong phenylephrine response is elicited. After evaluating the phenylephrine test results, in terms of the excised amount of conjunctiva and Muller muscle, Putterman and Urist recommended 9 mm resection in patients with mild eyelid elevation after phenylephrine use, and 7 mm in patients with increased eyelid height^(^2^)^. If the ptotic eyelid rises 2 mm after the phenylephrine test, Dresner recommends a 4 mm MMCR for every 1 mm of ptosis correction. Dresner also recommended an additional excision when the ptotic eyelid response to phenylephrine is <2 mm^(^3^)^. MMCR has also been shown to be successful in patients with a negative phenylephrine test^(^4^)^. MMCR is preferred because of the predictability of outcomes before surgery and preservation of the natural eyelid contour with the blink reflex^(^7^,^8^,^9^)^. To date, the mechanism of the beneficial effects of conjunctival mullerectomy has been widely debated. Marcet et al.^(^10^)^ conducted histopathological examination of cadaver specimens that underwent conjunctival mullerectomy and found conjunctiva and Muller muscle in all specimens. Thus, they suggested that conjunctival mullerectomy results in the shortening of the posterior lamellae, which causes advancement of the levator muscle and plication of the levator aponeurosis.

Although various posterior approaches are effective for ptosis repair, corneal injury, foreign body sensation, and granuloma formation that may be associated with suture material are potential postoperative complications. In addition, there is a concern that MMCR may lead to worsening dry eye, due to the possibility of injuring the adjacent accessory lacrimal glands and healthy conjunctiva^(^11^,^12^,^13^)^. Accessory lacrimal glands (glands of Wolfring and Krause) provide basal secretion of the aqueous layer in the tear film^(^14^)^. Krause glands are located in the upper conjunctival fornix and Wolfring glands are located in the upper border of the tarsus. Given Jordan’s work questioning the existence of essential tear flow, although Wolfring glands are closer to the resection site, their potential loss may be of little importance. Since some authors attribute up to 95% of tear secretion to the main lacrimal gland, this can probably overcome the loss of Wolfring glands in most patients^(^15^)^. Marcet et al.^(^10^)^ demonstrated preservation of the Krause glands in the upper conjunctival fornix and Wolfring glands in the upper tarsal border in exenterated orbits. Additionally, in MMCR, there is a loss of goblet cells that secrete the mucin layer of the tear film, depending on the amount of conjunctival tissue removed. Dailey et al.^(^16^)^ found no significant effect of upper eyelid ptosis repair by MMCR on tear production, as measured by the Schirmer test in 71 patients. In their study, the subjective dry eye symptoms transiently increased in the early postoperative period but often improved in the late follow-up period. Karabulut et al.^(^5^)^ found no decrease in tear production, as assessed by Schirmer’s test, and no significant dry eye in patients who underwent MMCR without tarsectomy In the present study, NIKBUT, OSDI, and OXFORD score values deteriorated significantly at 1 month after MMCR compared to the preoperative levels. This deterioration continued in the 3rd month after surgery, but there was a significant improvement in the 6th month after surgery. Similar to previous studies, our study also found that the subjective dry eye symptoms due to tear instability after MMCR improve over time. The worsening of NIKBUT, which occurs in the early period and is not permanent, indicates no significant loss in the accessory lacrimal glands. We think that these transient findings may be due to disruption of the tear aqueous/lipid balance due to postoperative inflammation. These results suggest that the excision of mucin-secreting conjunctival goblet cells during surgery is unlikely to significantly affect the tear film in the long term. This may be due to the adequacy of the remaining goblet cells to maintain normal tear stability.

Meibomian glands (MG); however, are sebaceous glands located in the tarsus parallel to each other and perpendicular to the lid margin. The number of MG in each eyelid varies between 15 and 25. These glands open at the lid margin at the skin-mucosal junction. They produce Meibum, which forms the lipid layer of the tear film and spreads to the ocular surface via the upper lid. Meibum acts as a surfactant and prevents evaporation of the aqueous component of the mucus-aqueous layer of the tear film^(^17^)^. Meibography is commonly used to evaluate MG morphology and MG changes. It has a high specificity and sensitivity in the diagnosis of MG dysfunction and dry eye. The 4-point subjective scale used to assess MG loss is observer-dependent. The ImageJ or Phoneix software digital classification system; however, provides an objective assessment of MG loss in cases with grade 4 or 5 subjective Meiboscores. In the present study, MG loss in patients who underwent MMCR was evaluated using the Phoenix meibography imaging software module^(^18^,^19^)^. A worsening in MGL and Meiboscor values was observed in the 1st and 3rd months after MMCR compared to the preoperative period. However, this deterioration improved to preoperative values in the 6th month after surgery. This shows that, unlike Fasanella-Servat surgery, MMCR does not entail any loss of tarsus, preventing any loss of lipid-secreting MG^(^1^,^2^)^. However, MMCR may cause minor trauma to MG because of the proximity of the removed tissue to the upper border of the tarsus, leading to tear instability. This may be the reason for the deterioration in MGL and Meiboscore occurring in the early period after MMCR in our study.

In conclusion, in this study, we observed no permanent MG loss at the 6th postoperative month in patients who underwent MMCR, as assessed by objective meibomyography measurements. Patients who underwent MMCR showed improvement in tear instability in the 3rd month after surgery. There was no permanent damage to the elements required for a healthy tear film. The deterioration in tear parameters observed in the first month after MMCR may predispose to the emergence of postoperative ocular surface complications. Therefore, topical lubricants should be administered in the early postoperative period after MMCR and should be continued for at least 3 months.

The retrospective study design and variability with respect to the amount of resected conjunctiva between patients are the limitations of our study. Controlled prospective studies are required to evaluate the effects of the amount of resected conjunctiva on tear parameters after MMCR.
